# Multi-Objective Optimization of a Wireless Body Area Network for Varying Body Positions

**DOI:** 10.3390/s18103406

**Published:** 2018-10-11

**Authors:** Łukasz Januszkiewicz, Paolo Di Barba, Sławomir Hausman

**Affiliations:** 1 Institute of Electronics, Lodz University of Technology, 90-924 Łódź, Poland; slawomir.hausman@p.lodz.pl; 2 Department of Electrical, Computer and Biomedical Engineering, University of Pavia, I-27100 Pavia, Italy; paolo.dibarba@unipv.it

**Keywords:** WBAN, wearable antenna, human body model, FDTD analysis, evolutionary optimization algorithms, computational electromagnetics

## Abstract

The purpose of this research was to improve the performance of a wireless body area sensor network, operating on a person in the seated and standing positions. Optimization-focused on both the on-body transmission channel and off-body link performance. The system consists of three nodes. One node (on the user’s head) is fixed, while the positions of the other two (one on the user’s trunk and the other on one leg) with respect to the body (local coordinates) are design variables. The objective function used in the design process is characterized by two components: the first controls the wireless channel for on-body data transmission between the three sensor nodes, while the second controls the off-body transmission between the nodes and a remote transceiver. The optimal design procedure exploits a low-cost Estra, which is an evolutionary strategy optimization algorithm linked with Remcom XFdtd, a full-wave Finite-Difference Time-Domain (FDTD) electromagnetic field analysis package. The Pareto-like approach applied in this study searches for a non-dominated solution that gives the best compromise between on-body and off-body performance.

## 1. Introduction

Wireless Body Area Networks (WBANs) are a lively field of research and development because they promise the long-awaited progress towards the widely-available monitoring of physiological parameters—vital signs—in healthcare, health and safety, military applications, and elsewhere [1]. This potential has been recognized by both research and engineering communities, resulting in efforts to standardize this class of wireless networks [2]. The standards establish a common framework for the development of WBAN applications, but many issues remain open for research and development, including the use of recognized optimization methods. Some research has been conducted into the improvement of WBANs, but mainly in the higher layers of the Open System Interconnection (OSI) model. For instance, in Reference [3] parameters such as payload length and the number of transmission retries were optimized and in Reference [4] the packet size was optimized. Research has also been conducted into the optimization of wearable antennas (e.g., Reference [5]). Nevertheless, one of the major and intrinsic difficulties with WBANs follows from the complex and unfavorable characteristics of the radio channel for both on-body and-off body links. There may be relatively high and variable path loss between some nodes, due in part to the presence of a human body: its posture and position relative to environmental elements. This problem is exacerbated during the outdoor operation of WBANs, when multipath propagation is weak and does not compensate for the high path loss of the direct node-to-node radio channel [6]. Much effort has been devoted to investigating path loss for various body positions, such as standing and sitting [7]. However, these have focused on certain predefined and fixed postures. In this paper, we formulate and solve a somewhat different, multi-objective multi-parameter optimization problem, i.e., the automatic optimization of node position on the user’s body. Our objective is twofold: to reduce the on-body path losses between nodes and to improve the systemic off-body channel loss (considering the directivity of the wearable antenna system).

The paper presents a different and more advanced approach in comparison to our previous work [8], in which we imposed a number of limiting design assumptions—for example, fixed positions for the head and arm nodes. The only degree of freedom was the placement of the chest node. The results were encouraging: a considerable decrease in the path loss between nodes using a single objective automated optimization procedure was achieved, based on evolutionary computing. The current paper has a more ambitious aim. Minimizing a multi-objective function, which simultaneously depends on a set of design variables, is a vitally important problem in many fields of engineering. It is part of many optimization routines which are used widely to search for possible improvements in already existing engineering designs, often developed by heuristic methods or inaccurate design formulas. This also refers to radio communication, where antenna design or wireless system performance can be improved—for instance, by reducing transmit power and battery energy consumption [9].

The design of electromagnetic devices and systems typically requires controlling several aspects of their performance, resulting in multiple design criteria that give rise to multi-objective design problems [10,11,12,13,14,15,16]. To cope with a vector of design criteria, a fully Pareto-like formulation or a preference-function approach can be used. In the former case, the set of best compromise solutions (also known as non-dominated solutions) has to be identified. In contrast, in the latter case, the single-objective algorithms can be exploited, especially those belonging to the evolutionary class, which have proven to be cost-effective and global-optimum oriented. In the research presented in this paper, we used a Pareto optimization algorithm. In the performance optimization loop for wireless body area networks (WBANs), we used a full-wave numerical electromagnetic code with a numerical model of the human body to calculate the objective function components, such as the path loss or the off-body radiation pattern. Standard body models are available in electromagnetic simulation programs, which are made for a human body in the standing (or lying) positions. It is possible to reposition these models into different poses with specialized programs (e.g., Remcom VariPose [17]), but because of the lack of a suitable programming interface, the software cannot be run automatically within an optimization loop. Our optimization procedure is currently adapted for two body postures: sitting and standing. For this purpose, two simplified models of a human body consisting of a set of cylinders are proposed. These were generated swiftly and automatically in Remcom XFdtd [18], which is a commercial package implementing full-wave Finite-Difference Time-Domain (FDTD) electromagnetic simulator, for each optimization step using the available scripting language. Our approach is flexible because it can be also used in the future for other body postures.

The major goal of our current study was to develop a strategy for the automatic optimization of the location of WBAN nodes for varying body positions of the user. We used the Remcom XFdtd simulation program in this research, which utilizes the FDTD method, and the numerical human body model which we have elaborated. The model was verified by us experimentally [19]. We found that simulation results obtained for the 2.4 GHz ISM frequency band with the heterogenic NMR Hershey human body model in XFdtd are similar to the results of measurements obtained for a human subject and for the simplified 6-cylinder phantom.

## 2. Wireless Body Area Sensor Network

**Figure 1:**
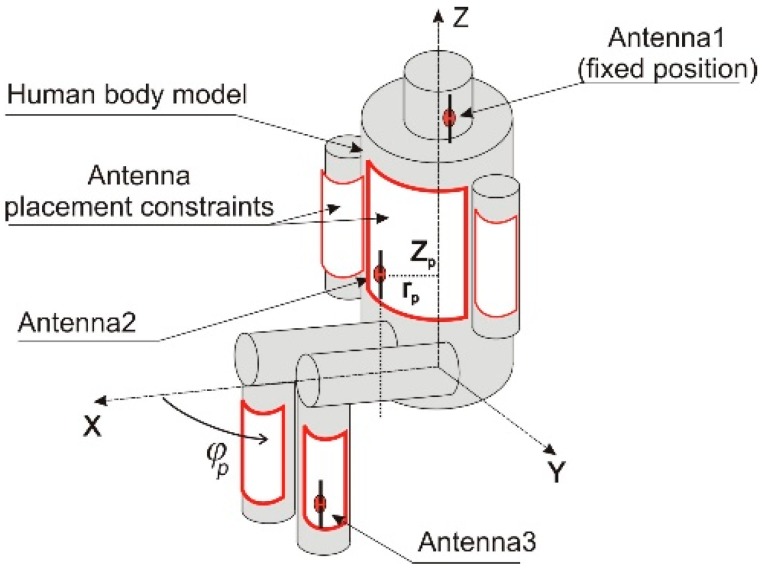
The wireless body area sensor network on the simplified cylindrical human body model.

The WBAN analyzed in this research, which we designed originally for monitoring the vital signs of firefighters, consists of miniature nodes which use transceivers operating in the 2.4 GHz ISM (Industrial, Scientific, and Medical) band. The studied network consists of three nodes which measure certain physiological parameters of the human body and transmit the data both to each other (on-body channels) and to a remote node located away from the body (off-body channel). In our computer simulations, all nodes use half-wave dipole antennas tuned to 2.4 GHz. We designed dipoles with shortened geometry to match the antenna port impedance to 50 Ω, which resulted in a total length of 54 mm, a conductor diameter of 1 mm, and a feeding point gap of 1 mm. One node (connected to antenna 1; see Figure 1) contains a pulse-oximeter to measure blood oxidation and heart rate. The sensor is attached to the ear-lobe and the node, therefore, has to be located close to the ear. It was assumed that this node has a fixed position on the left side of the head. Node 1 has a low-power transceiver and a miniature battery, which enables data transmission only to the two other nodes located on the user’s trunk, constituting an on-body network.

**Figure 2:**
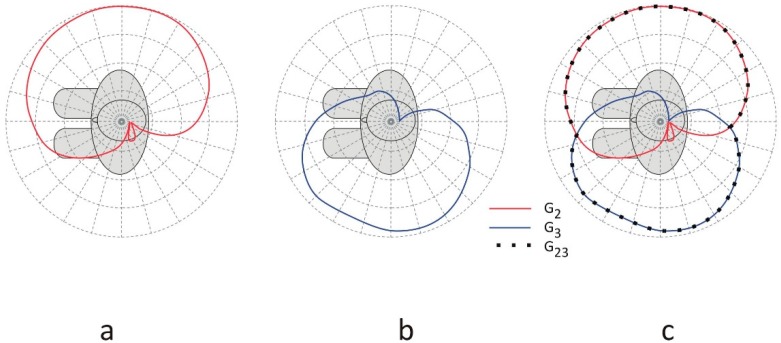
The off-body communication scenario: (**a**) transmission for antenna 2 with gain G_2_; (**b**) transmission for antenna 3 with gain G_3_; (**c**) transmission for both antennas 2 and 3 with the resultant gain G_23_. It should be noted that in any given direction, G_23_ is not the arithmetic sum of G_2_ and G_3_ but the greater of the two, because the two transceivers operate at the same frequency but in different time slots. In that sense, this is not a two-element antenna array with the superposition of radiation patterns but a switched pattern antenna.

The trunk node, located on the user’s chest, measures the respiratory rate as well as the temperature and humidity of the firefighter’s clothes (antenna 2 in Figure 1). Another node performs the function of a pedometer and is placed on the user’s calf (antenna 3 in Figure 1). Both the trunk and leg nodes can receive data from the pulse-oximeter and from each other (on-body transmission). Nodes 2 and 3 are also capable of transmitting measurement data to the remote receiver (off-body transmission). Figure 2 presents the off-body transmission scenario. The received power in this link depends strongly on the wearable antenna gain, especially in an outdoor environment, in which the multipath effect is very small [6]. Due to the influence of the human body on the wearable antenna radiation pattern for a wide range of radiation angles, the antenna gain can be very low. Depending on the position of the antenna on the human body, the antenna gain can be lower than −10 dBi for a sector wider than 100° in the horizontal plane [20]. When the propagation path between the human body and the remote receiver falls into the low gain zone of the antenna radiation pattern, the power of the received signal can drop below the receiver’s sensitivity.

Figure 2 also presents the differences in the radiation patterns of the wearable antennas. Antenna 2 is located on the right arm and antenna 3 is placed on the left side of the calf. In this case, the radiation of antenna 2 (with gain G_2_) presented in Figure 2a covers the right side of the body with very low gain on the left side, while the radiation of antenna 3 presented in Figure 2b (with gain G_3_) is directed to the left side and does not cover the right side of the body completely. In the proposed system, both transceivers 2 and 3 operate at the same frequency, but different time slots are utilized for transmitting the measurement data from the body to the remote receiver in the off-body link. The remote receiver can select the stronger signal of the two that come from nodes 2 and 3, as presented in Figure 2c. This two-antenna transmission scenario improves the off-body communication performance because the minima of the radiation pattern of one antenna can be filled in by the radiation from the other antenna, depending on the positions of the antennas on the human body. In Figure 2c, the gain of the wearable antennas of transceivers 2 and 3 operating in different time slots is defined as G_23_(*ϕ*) = *sup*(G_2_(*ϕ*), G_3_(*ϕ*)). The resultant gain G_23_ is equal to the greater of the two, G_2_ or G_3_*,* over the angles in the horizontal plane, meaning that the receiver will always select the strongest available signal to detect. Therefore, it should be noted that G_23_ is not the sum of G_2_ and G_3_.

**Figure 3:**
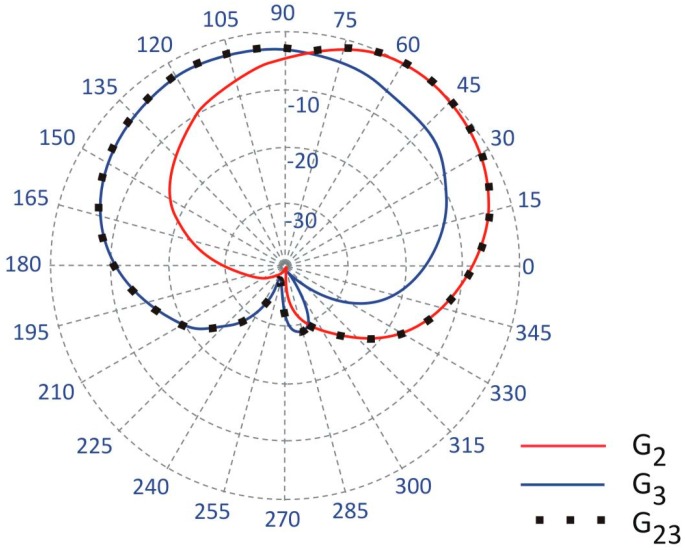
The radiation patterns of antennas in the off-body link obtained for their initial positions (*ϕ*_2_ = 65°, *z*_2_ = 0.32 m, *ϕ*_3_ = 107°, *z*_3_ = 0.21 m), normalized to the maximum gain value equal to 6.5 dBi. In this case, there is no significant improvement in the performance because the radiation patterns strongly overlap.

This two-antenna switched-pattern approach presented also in [20], can give a better system performance than a two antenna array operating with a single transceiver, a power splitter, and two waveguides. The wearable two-antenna array would suffer from a radiation pattern with deep interference minima which could cause a signal drop, particularly in outdoor environments [21]. The time-diversity in off-body transmission from the two antennas located on the opposite sides of a body reduces the minima in the resultant horizontal pattern gain. Our solution can improve the link reliability in outdoor systems, depending on the particular position of the antennas with respect to the body [20]. To obtain a stable and reliable off-body link irrespective of the free movement of the user, the wearable antennas should have a quasi-omnidirectional pattern. In two-antenna systems, this can be achieved by the complementarity of the radiation patterns for antennas 2 and 3. Randomly selecting the locations of the antennas can lead to a poor off-body link, as may be observed in Figure 3, where for angles 210–315°, the gain of both antennas is below −20 dB. Because the antennas are close to the human body, which influences their radiation properties, they have to be positioned appropriately. This was our motivation for applying an automated optimization procedure to the placement of the antennas.

In the optimization process, the geometrical coordinates of nodes 2 and 3 on the trunk surface are the design variables. They have an influence on both the path loss in the on-body link and the radiation pattern in the off-body link. However, while the locations of the antennas on opposite sides of the body result in a desirable quasi-omnidirectional diversity pattern, at the same time, the on-body transmission suffers from high signal attenuation, introduced by the human tissues. The goal was, therefore, to optimize the performance of the off-body link while maintaining good performance in the on-body transmission channel.

**Figure 4:**
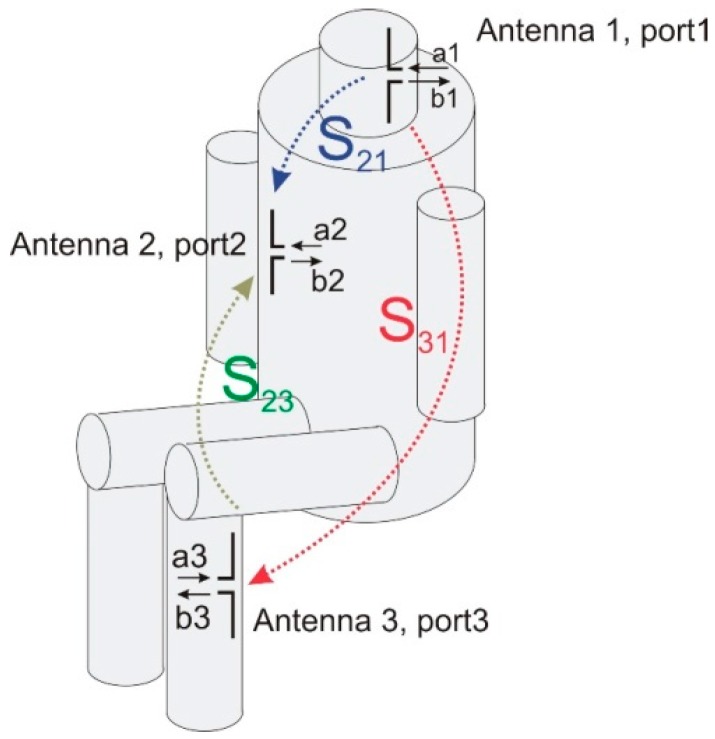
The scattering parameters defined with reference to the on-body antenna signals.

The on-body performance of the network was evaluated by scattering matrix parameters defined for a 3-port network, where each port refers to an antenna port, as presented in Figure 4. The scattering matrix is defined by Equation (1).

(1) [B]=[S]·[A]

where:

B: the vector of output signals
S: the scattering matrix
A: the vector of input signals.

In the case of a network that consists of three antennas, the scattering matrix S is given by Equation (2).

(2) $$\left[\begin{array}{c} b_{1}\\ b_{2}\\ b_{3} \end{array}\right]=\left[\begin{array}{ccc} S_{11} & S_{12} & S_{13}\\ S_{21} & S_{22} & S_{23}\\ S_{31} & S_{32} & S_{33} \end{array}\right]\cdot \left[\begin{array}{c} a_{1}\\ a_{2}\\ a_{3} \end{array}\right]$$

In the case of the network considered in this paper, the matrix is diagonally symmetrical. To characterize the attenuation of the signal between the antennas, only three parameters are needed. In what follows, only the parameters $S_{21},S_{31},S_{23}$ are considered.

## 3. Simplified Body Model for the Sitting Position

**Figure 5:**
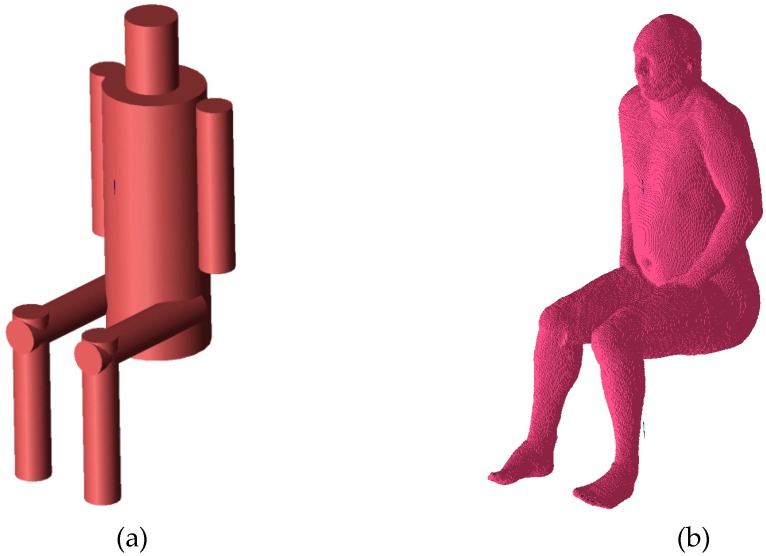
The human body models: (**a**) simplified-cylindrical; (**b**) anthropomorphic (multitissue).

Various human body models and full-wave electromagnetic codes can be used to evaluate the performance of on-body and off-body wireless channels. Simplified cylindrical models of the human body are particularly useful for modeling different poses [22,23] because the positions of the body parts can be easily modeled by translation and rotation of the cylinders. Cylindrical models are also convenient when the antenna position is variable but its distance to the body surface is fixed. This is because the position of the antenna can be controlled easily with two coordinates in a cylindrical coordinate system. In the case of anthropomorphic models, where the body shape is complex (non-cylindrical), maintaining a fixed distance between the antenna and body (e.g., the average across the antenna surface, the minimum, or another distance metric) is difficult. The results of path loss simulations for the on-body channel obtained with the simplified cylindrical model are satisfactorily similar to those obtained with a heterogeneous model [23]. The geometry of the simplified model was adjusted to approximate the shape of the heterogeneous model, as shown in Figure 5.

**Figure 6:**
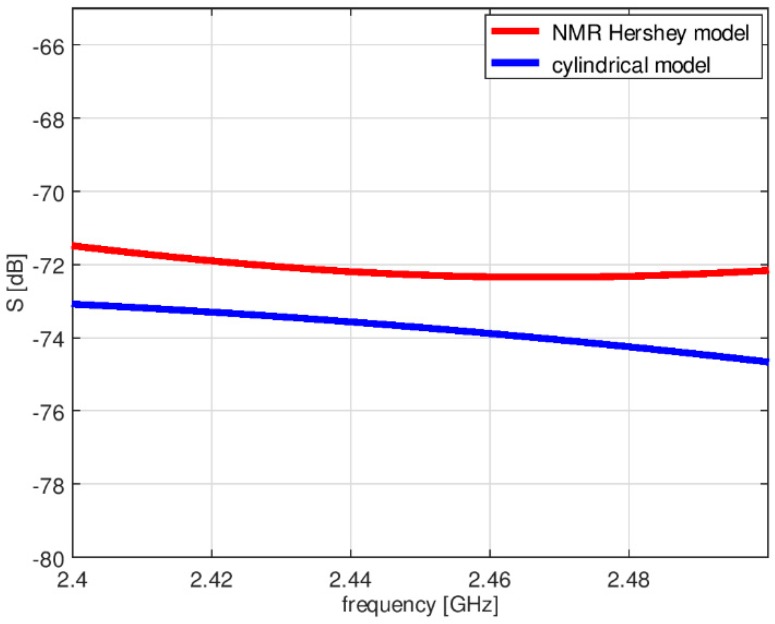
The system loss between the chest antenna (2) and leg antenna (3) (see Figure 4) obtained using the heterogeneous model and the simplified model.

**Figure 7:**
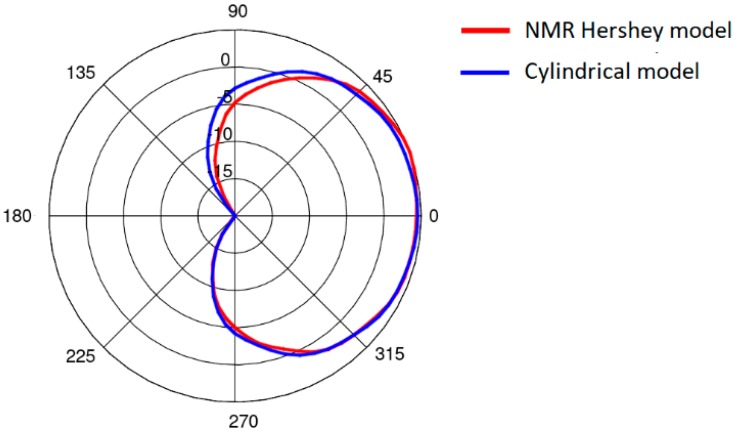
The on-chest antenna (antenna 2) radiation pattern in the horizontal plane obtained using the heterogeneous model and the simplified model.

**Figure 8:**
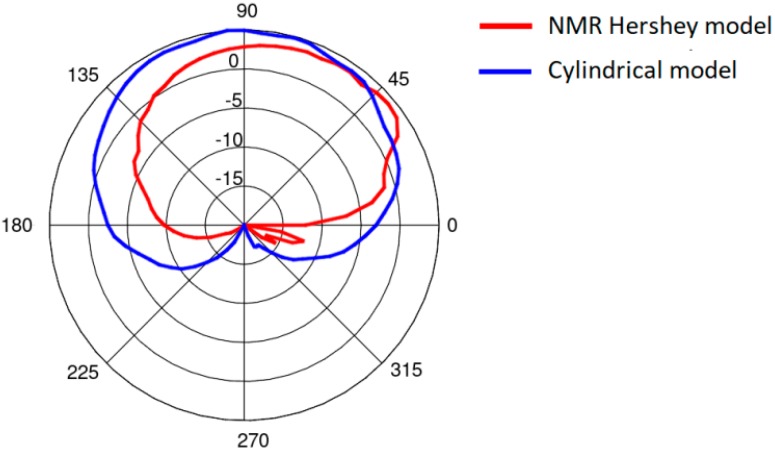
The on-leg antenna (antenna 3) radiation pattern in the horizontal plane obtained using the heterogeneous model and the simplified model.

The simplified model was verified with computer simulations carried out using both the simplified and the heterogeneous models. The heterogeneous model was modified in the Remcom VariPose [17] program to obtain a sitting position. The results of measurements obtained for the cylindrical model are similar to those obtained with a human subject [22,23]. Figure 6 shows a comparison of the path loss between the chest antenna (G_2_) and the leg antenna (G_3_) computed using XFdtd with the heterogeneous model and the simplified model. The maximum difference is around 3 dB. Figure 7 shows a very good agreement between the on-chest antenna (G_2_) radiation patterns calculated using the two models. A significantly worse agreement (up to 12 dB difference) can be observed in Figure 8 for the on-leg antenna gain. This discrepancy will be investigated in our future research since for the purposes of the present study the approximation of the antenna directivity pattern is good enough to test our optimization strategy. The simplified cylindrical model was therefore chosen for further optimization of WBAN antenna placement. Its geometry can be synthesized and integrated with the antenna’s geometry within the XFdtd scripting language with very low computational effort in the optimization loop. The geometry allows for the convenient movement of the antennas around the parts of the body in the cylindrical coordinate system axis of the trunk or leg, maintaining a constant distance between the antennas and the body.

## 4. Optimization Problem

### 4.1. Formulation

*The problem with the optimal placement of wireless nodes can be posed as follows: given a sensor located at a fixed point on the head of a human body, find locations (g) for two other wireless sensors, which should be placed on the trunk or arms and on one leg for operation in the standing and sitting positions with twofold goals: minimize the on-body channel path loss represented by the objective function* f_1_*(g) (3) and simultaneously maximize the off-body gain represented by the objective function* f_2_*(g) (4), in the frequency band of 2.4 GHz*.

The following definitions will be used in the analysis:

$g\in {ℜ}^{4}$: the design vector (i.e., the set of four cylindrical coordinates identifying the positions of the two wireless sensors: on the trunk and on one of the legs)
Ω_*g*_: the set of admissible positions
B: the 2.4 GHz band
*k* = 1: the standing posture index; *k* = 2: the sitting posture index
$G_{1+2}=G_{1}\cup G_{2}$: the off-body directivity pattern (i.e., the maximum of individual radiation patterns G_1_ and G_2_ in a given direction).

Starting from a feasible solution *g*_0_ in Ω_*g*_, the following f_1_ objective is to be maximized:

(3) $$f_{1}\left(g\right)=\underset{k}{\inf}\left[S^{\left(k\right)}\left(g\right)\right]\;\;,\;\;k=1,2\;\;,\;\;g\in \Omega_{g}$$

where

$$S^{\left(k\right)}=\underset{f}{\inf}\left[S_{21}^{\left(k\right)}\left(f\right),\;\;S_{31}^{\left(k\right)}\left(f\right),\;\;S_{32}^{\left(k\right)}\left(f\right)\right];\;\;\;k=1,2;\;\;\;f=\left(2400+l\cdot 0.440\right)\left[\mathrm{MHz}\right];\;\;l\in \left[0,226\right]$$

and, simultaneously, the following f_2_ objective is to be maximized:

(4) $$f_{2}\left(g\right)=\underset{k}{\inf}\left|G_{1}^{\left(k\right)}\left(g\right)\cup G_{2}^{\left(k\right)}\left(g\right)\right|\;\;,\;\;\;k=1,2\;\;,\;\;g\in \Omega_{g}$$

The values of the scattering matrix elements S_21_(f), S_31_(f), and S_32_(f) are calculated within the frequency band of interest 2.4–2.5 GHz with an interval of 440 kHz—i.e., for 227 values of f. These values are elements in the Fourier Transform of the time-domain response to wideband excitation obtained from a single run of the FDTD analysis. From Equations (1) and (2), it appears that our optimization problem is characterized by four design criteria, i.e., the path loss S and gain G_1+2_ for each posture *k* = 1, 2, depending on the set of four design variables. Maximizing f_1_ and f_2_ over *k* gives rise to a min-max formulation. It is, therefore, possible to reduce the number of independent objective functions to just the (f_1_,f_2_) pair. A bi-objective optimization problem is thus formulated:

Starting from a guess solution, find the values of a four-dimensional design vector (coordinates of two wireless nodes on the user’s body) yielding a Pareto-optimal solution, i.e., a compromise point trading off the on-body path loss and the off-body radiation pattern.

### 4.2. The Algorithm

An evolutionary algorithm of the lowest order, originally proposed in Reference [24], was used for the optimization. The basic underpinning principle is to accept a new candidate solution only if it dominates all the objectives in a Pareto sense. Accordingly, let *m* be the parent solution and let *x* be an offspring originated by perturbing *m* based on a Gaussian probability density. The selection criterion is such that offspring *x* is accepted if and only if the following relationship (Pareto-like selection) holds true:

(5) $$f_{1}\left(x\right)>f_{1}\left(m\right)\;\mathrm{and}\;f_{2}\left(x\right)>f_{2}\left(m\right)$$

i.e., if *x* improves both objective functions with respect to *m*. The exploration capacity of the algorithm is substantial: when Equation (3) is fulfilled, the search radius is increased, i.e., the region in which the subsequent offspring may occur is broadened in order to find an even better improvement. This feature prevents the trajectory from becoming trapped in a local optimum, so the algorithm is considered to be global-optimum oriented. The computational cost is low because in each iteration only two solutions are necessary, i.e., the current—or parent—solution *m* and the relevant perturbation—or offspring *x*. The selection operator (3) acts on the (*m*, *x*) pair. The convergence property of the algorithm relies on controlling the standard deviation which is associated with each variable: the relevant Gaussian distributions tend to be deterministic as long as the current solution saturates the possible improvement of the objective functions.

**Figure 9:**
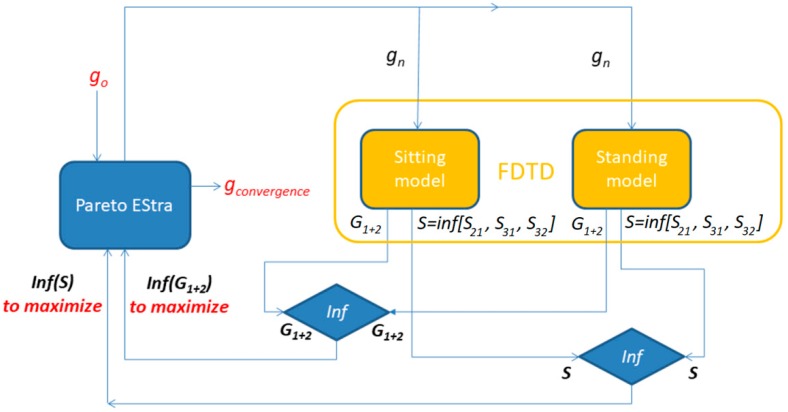
The flowchart of the computational scheme.

The whole computational scheme links the optimization algorithm with the FDTD analysis for objective function computation. The relevant flowchart is shown in Figure 9. The optimization routines are implemented in Matlab while the evaluation of S parameters and antenna gain is performed in the Remcom XFdtd software ver. 7.6.0.2.

Starting from the initial guess solution, an optimization trajectory is originated, eventually leading to the approximation of a non-dominated solution in the objective space. As a side product, the set of solution points generated during the search forms a cloud, sampling the objective space.

In order to keep the computational cost moderate, the basic version of P-EStra, which we used in this study, exploits the evolution of a single individual, i.e., one parent is used to create one offspring, which is known as the (1 + 1) evolution strategy. As a consequence, the algorithm converges to a single non-dominated solution g* which improves over the starting solution *g*_0_ with respect to both objective sub-functions f_1_(*g*) and f_2_(*g*). Even if the Pareto front is not explicitly computed, it should be emphasized that the optimized solution g* belongs to the subset of the front dominating the initial solution *g*_0_. On the other hand, however, due to the stochastic behavior of the algorithm, the location of the optimized solution g* along the relevant Pareto front is out of control. Our choice of the (1 + 1) evolution strategy follows from the high computational cost of the evaluation of the objective sub-functions f_1_(*g*) and f_2_(*g*) which involves time-consuming full-wave FDTD simulations. Using a computer equipped with two NVIDIA TESLA C2075 accelerator card with two multicore Graphical Processor Units (448 CUDA cores each), the time necessary to calculate the objective function values was in the range of 25 minutes in each iteration.

## 5. Simulation and Optimization Results

**Figure 10:**
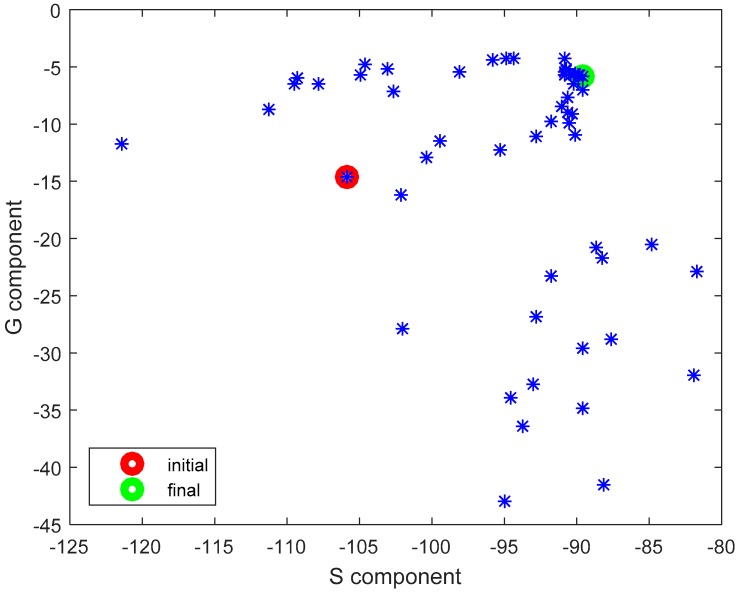
The history of the optimization process in the objective function space.

**Figure 11:**
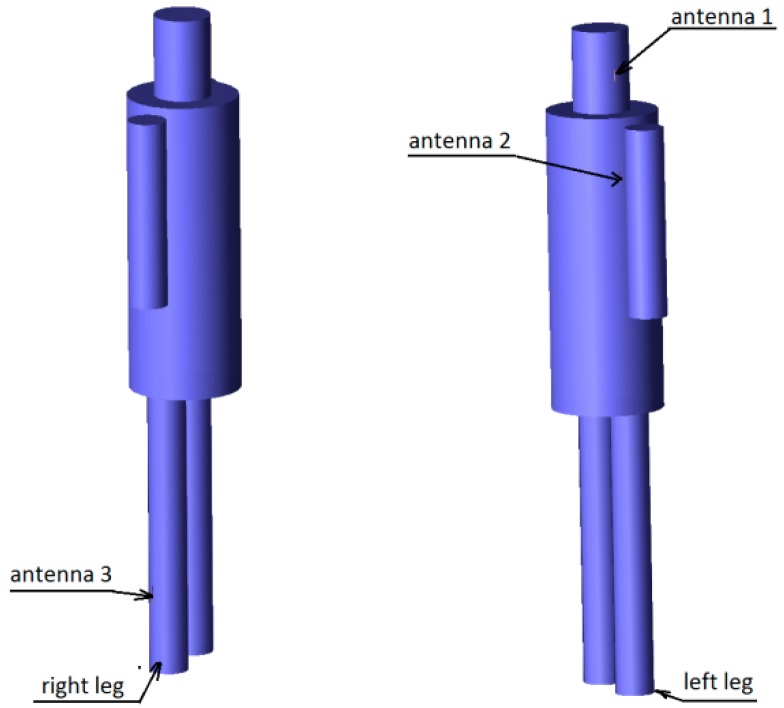
The initial placement of antennas on the standing model.

**Figure 12:**
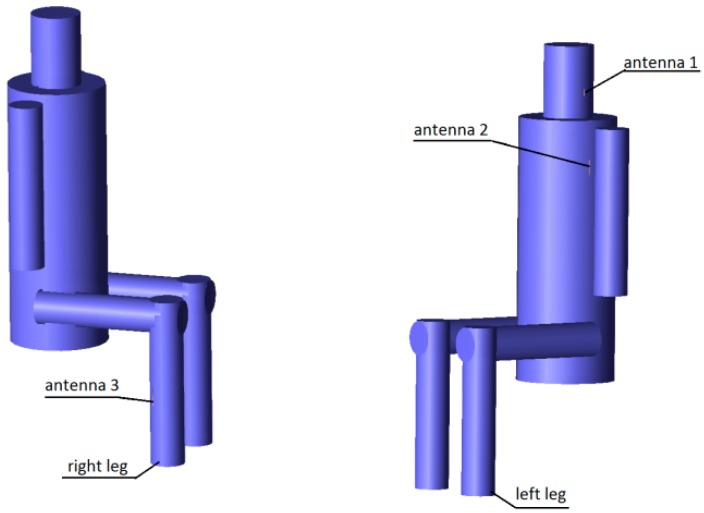
The initial placement of the antennas on the sitting model.

**Figure 13:**
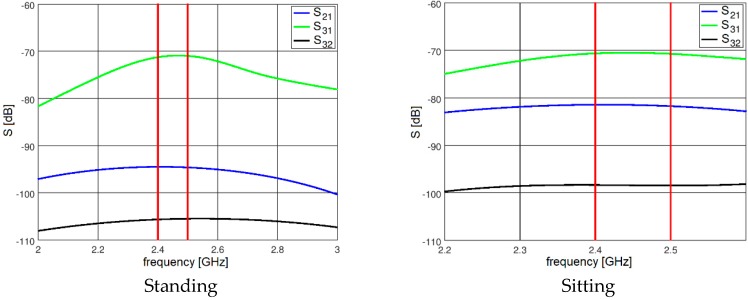
The initial S parameters.

**Figure 14:**
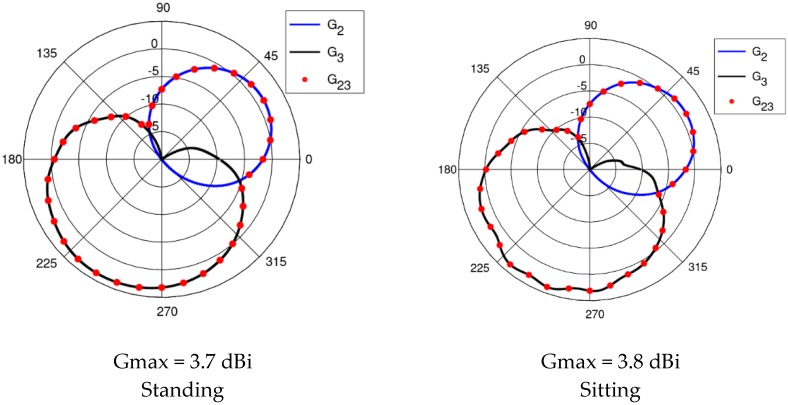
The initial radiation patterns.

**Figure 15:**
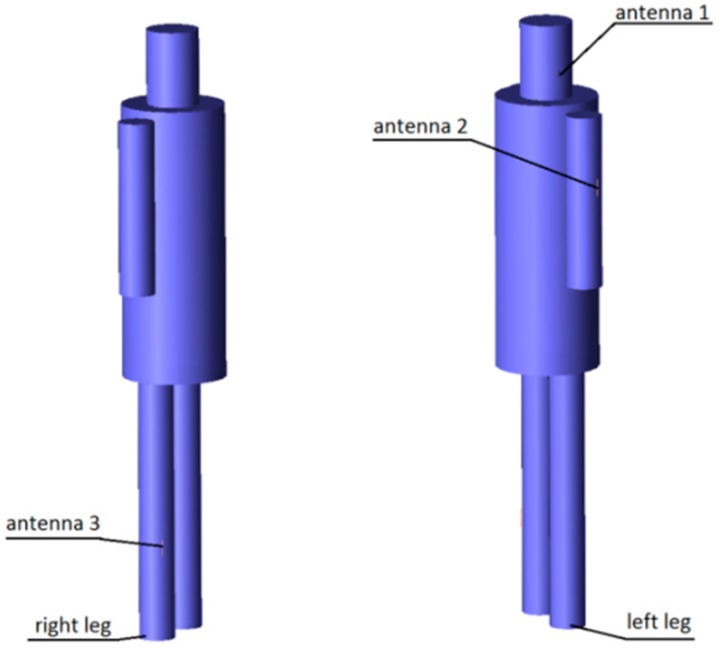
The final placement of the antennas on the standing model.

**Figure 16:**
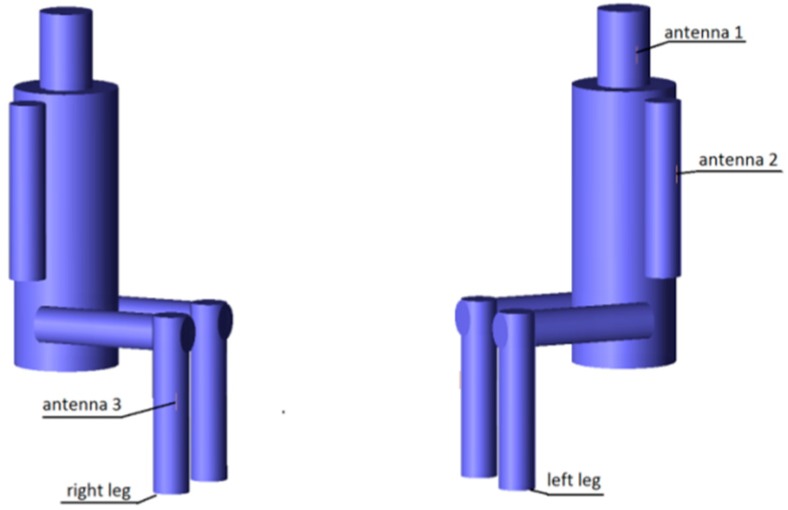
The final placement of the antennas on the sitting model.

**Figure 17:**
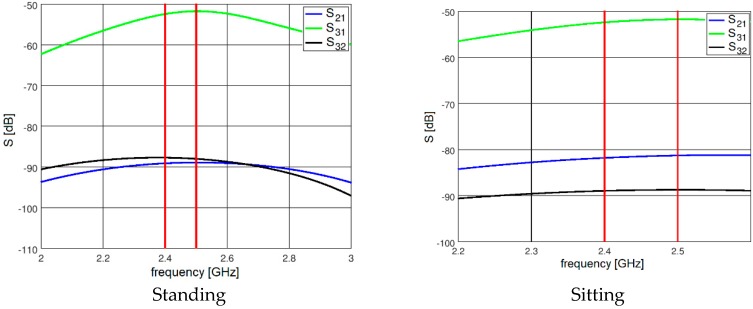
The S parameters for the final solution.

**Figure 18:**
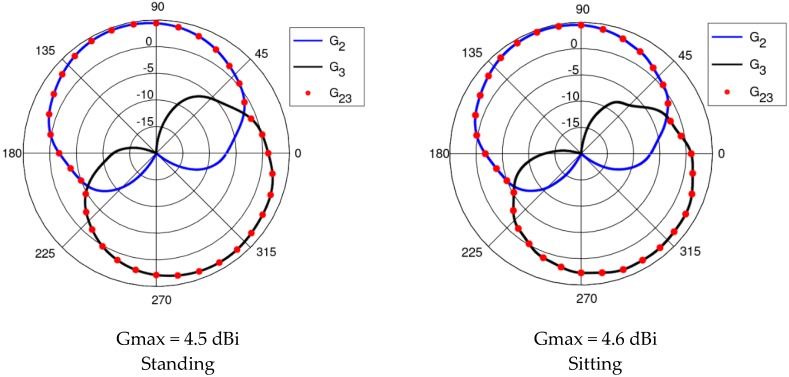
The radiation patterns for the final solution.

The results presented and discussed in this section were obtained for a three-antenna system as described in Reference [20]. To make the results of our investigation more general, a very simple antenna was modeled in this study with the radiation pattern independent of rotation around the *z*-axis. The antennas are dipoles placed in a vertical position matched to operate at 2.45 GHz. The initial placement of the antennas was defined by the positions of the pockets on the firefighter’s uniform, which were originally considered for the wireless sensors. The coordinates of the second antenna were *ϕ*_2_ = 1.4 rad and *z*_2_ = 1.03 m. The coordinates of the third antenna were *ϕ*_3_ = −2 rad and *z*_3_ = 0.23 m. Figure 10 shows the history of the optimization process in the objective function space. The initial values for the objective function components were S_start_ = −105.8, G_start_ = 0.034, where S_start_ = inf[S_31_,S_21_,S_23_], G_start_ = G_1+2_ before optimization. The final values were S_stop_ = −89.8, G_stop_ = 0.27, where S_stop_ = S_21_, and G_stop_ = G_1+2_ after optimization. Both components were significantly increased (improved). This solution was achieved in 55 iterations which lasted approximately 24 h, most of which was taken up by the time-consuming computation of the objective function components G and S. Figure 11 and Figure 12 present the initial positions of the antennas for the standing and sitting positions, respectively. These positions resulted in the scattering matrix values (as a function of frequency) shown in Figure 13 (for the standing and sitting positions) and the radiation patterns in Figure 14 (for the standing and sitting positions). The final coordinates of the antennas were *ϕ*_2_ = 2.26 rad, *z*_2_ = 0.94 m, *ϕ*_3_ = −0.94 rad, and *z*_3_ = 0.28 m, respectively, as shown in Figure 15 and Figure 16. The final positions resulted in the scattering matrix values (as a function of frequency) shown in Figure 17 (for the standing and sitting positions) and the radiation patterns in Figure 18 (for the standing and sitting positions).

**Figure 19:**
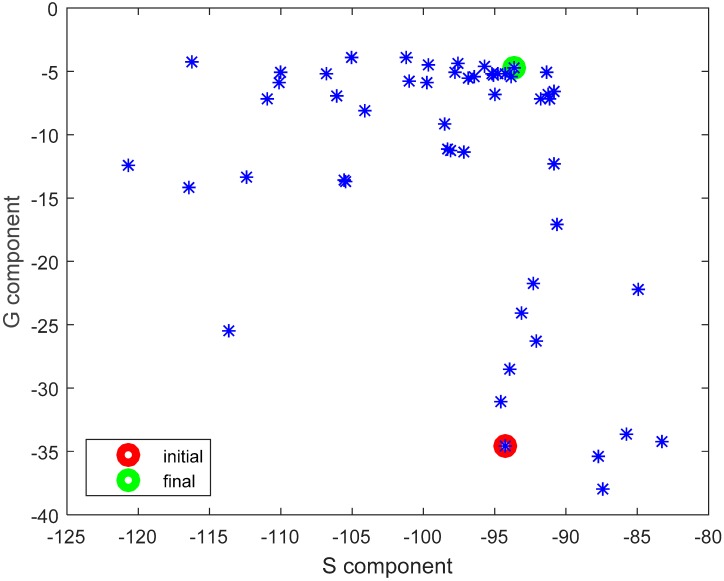
The history of the optimization process (2^nd^ case) in the objective function space.

An additional run of our multi-objective optimization process was performed from a different starting point. This time the initial placement of the antennas was intentionally configured in such a way that should result in relatively low path-loss in the on-body communication scenario. Antennas 2 and 3 were located relatively close to each other, on the same side of the body (on the front). The aim was to check whether our algorithm could improve the G component without significantly deteriorating the S component. The initial coordinates of the antennas were *ϕ*_2_ = 0 rad, *z*_2_ = 1.06 m, *ϕ*_3_ = 0 rad, and *z*_3_ = 0.18 m while the initial values of the objective components were S_start_ = −94.2, G_start_ = 0.00035, where S_start_ = inf[S_31_,S_21_,S_23_], G_start_ = G_1+2_ before optimization. The final values were S_stop_ = −93.58, G_stop_ = 0.337, where S_start_ = inf[S_31_,S_21_,S_23_], G_start_ = G_1+2._ The final coordinates were *ϕ*_2_ = 1.97 rad, *z*_2_ = 0.92 m *ϕ*_3_ = −1.53 rad and *z*_3_ = 0.23 m. Once again, both components were improved. The increase in the G component was particularly substantial. This optimization loop lasted 54 iterations and 24 h. The history of the process is presented in Figure 19, along with the cloud of intermediate solution points. It can be observed that even with significantly different starting points, the final solutions from our two optimization runs are similar with respect to the objective function values.

After optimization, the value of gain G_1+2_ for any azimuth and any of the two body postures (standing or sitting) takes on values that are always greater than around −5 dB, which can be regarded as a satisfactory result, considering the radiation patterns of the individual antennas (dipoles near the human body). This should enable the implementation of off-body communication. The worst value we obtained for on-body communication path loss was 93 dB. Assuming a hypothetical IEEE 802.15.6 transceiver with a −98 dBm sensitivity (typical for existing ZigBee transceivers operating in the 2.4 GHz band, for instance, Texas Instruments CC2520) and a maximum transmission power of 0 dBm (5 dBm for CC2520), the link budget is positive, albeit with a low margin of 5 dB. Given these results, we conclude that our methodology for the WBAN physical layer optimization is successful.

## 6. Conclusions and Future Work

### 6.1. Conclusions

In this paper, we have presented and discussed a methodology for optimizing a three-node WBAN design (node placement) with both on-body and off-body communication. Under this frame, the proposed approach lays the groundwork for a more general method for finding the optimal design of any WBAN configuration. The systematic exploitation of Pareto-like optimality is made possible by evolutionary computing linked with the FDTD full-wave electromagnetic simulation of antenna and radio channel performance. This computational method facilitates the search for innovative designs because it is able to identify non-trivial solutions, i.e., solutions improving the prototype performance with respect to all design criteria, which is difficult using the traditional trial-and-error approach. Specifically, in the research reported in this paper, we exploited our methodology to improve the operation of a WBAN on a human body in both sitting and standing positions by searching for the best configuration of the nodes. Because we evaluate two objective criteria (multi) for two (many) body positions, the method can be qualified as many-multi-optimization. Our numerical experiments revealed significant improvements with respect to both design criteria, i.e., on-body and the off-body channel performance.

### 6.2. Future Work

The assessment of our methodology presented in this paper is based on a small number of WBAN nodes (i.e., 3), with no need for routing. Nonetheless, our many-multi-optimization engine could be applied to a case with more nodes, further body positions, and additional design criteria. In future work, we, therefore, plan to apply our methodology in a more complex case with a number of nodes, involving multi-hop topologies and routing algorithms. In particular, we will consider energy efficiency, which is an area of great research interest, as one of the improvement criteria. However, whereas most of the many studies in the literature concentrate on the routing algorithms themselves, we will attempt to formulate and solve the node placement optimization problem using one of the existing efficient routing algorithms. Increasing the number of nodes will require greater computational effort since a larger number of scattering matrix elements *s*_ij_ will need to be evaluated. The routing simulation will be performed for the placement of each candidate node during the optimization process. 

A further focus of our future investigations will be the optimization of wireless networks operating in the ISM 5.8 GHz band as well as in the 3.6 GHz band allocated for the 5G (Fifth Generation) of wireless communication systems. For this purpose, we will analyze the performance of the network with different wearable antennas instead of simple dipoles.
